# Design of MOF-Derived Cd_1–*x*_Zn_*x*_S Solid-Solution Photocatalysts and Evaluation of Hydrogen Production by Photoreforming

**DOI:** 10.1021/acsomega.6c01944

**Published:** 2026-05-18

**Authors:** Yusei Kumai, Yuya Takekuma, Morio Nagata

**Affiliations:** † Department of Industrial Chemistry, Graduate School of Engineering, 26413 Tokyo University of Science, 6-3-1 Niijuku, Katsushika-ku, Tokyo 125-8585, Japan; ‡ Department of Industrial Chemistry, Faculty of Engineering, Tokyo University of Science, 6-3-1 Niijuku, Katsushika-ku, Tokyo 125-8585, Japan

## Abstract

The photoreforming of low-value biomass in alkaline aqueous media is a promising route for sustainable hydrogen production because it couples hydrogen evolution with the utilization of abundant organic resources. In such systems, sulfide photocatalysts are attractive because of their relative stability and favorable hydrogen evolution activity; however, their hydrogen production performance remains insufficient. To address this limitation, Cd_1–*x*_Zn_*x*_S solid-solution photocatalysts were synthesized using mixed-cation metal–organic frameworks (MOFs) precursors as a platform for precursor design. A series of Cd–Zn mixed MOFs with different Cd/Zn ratios were prepared using terephthalic acid as the organic linker and converted into the corresponding sulfides by sulfidation. Among the samples, CdZnS-73 showed the highest hydrogen production activity during photoreforming in an alkaline aqueous solution containing α-cellulose under simulated sunlight irradiation, with a hydrogen production rate 7.2 times higher than that of MOF-derived CdS. Furthermore, MOF-derived CdZnS-73 exhibited 9.2 times higher activity than a CdZnS sample synthesized without an MOF precursor. This enhanced activity is likely associated not only with solid-solution formation but also with local structural distortion resulting from the MOF-derived synthesis route. These results demonstrate that mixed-cation MOFs are effective precursors toward the design of highly active sulfide photocatalysts for hydrogen production via alkaline biomass photoreforming.

## Graphical abstract

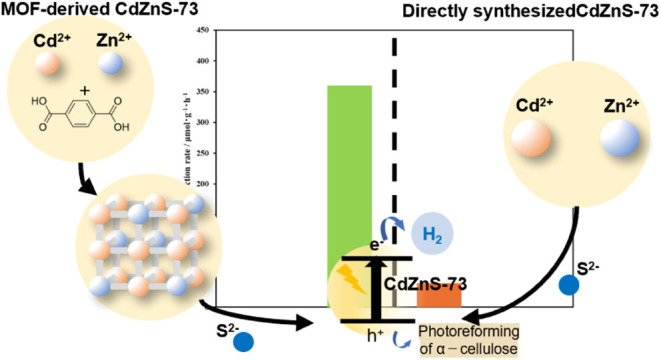

## Introduction

Fossil fuel depletion and climate change are major global challenges. Hydrogen is considered a promising next-generation energy carrier because of its high energy density and carbon-free combustion. However, the majority of hydrogen is still produced from fossil fuels, resulting in carbon dioxide emissions. Therefore, sustainable hydrogen production methods are urgently needed. Since the first report in 1972, photocatalytic hydrogen production has attracted considerable attention as a potential alternative.^1–5^ Photocatalytic overall water splitting has been widely investigated as a direct means of converting solar energy into chemical energy. In parallel, photocatalytic hydrogen production coupled with thermodynamically favorable oxidation reactions (Δ*G* < 0) has also attracted considerable attention. Although such systems do not strictly achieve net solar energy storage, they offer practical advantages because the oxidation of sacrificial reagents is both kinetically and thermodynamically easier than water oxidation. Consequently, they can increase the hydrogen evolution rate while suppressing photocorrosion, which is a major cause of catalyst deactivation in sulfide photocatalysts. However, conventional sacrificial reagents, such as methanol,^6^ triethanolamine,^7,8^ and sodium sulfide,^9,10^ are industrially valuable chemicals and can be costly, making them less suitable for large-scale and sustainable applications.

For this reason, increasing attention has recently been directed toward coupling hydrogen production with the photoreforming of abundant and low-value organic resources.^11,12^ Among these, lignocellulosic biomass, which is mainly composed of cellulose, hemicellulose, and lignin, stands out because it is abundant and renewable; additionally, it can be decomposed under alkaline aqueous conditions.^13–17^ In these systems, hydrogen evolution may involve the oxidation of cellulose itself and/or soluble low-molecular-weight products generated from alkaline hydrolysis, depending on the reaction conditions. In such alkaline photoreforming systems, sulfide photocatalysts are important owing to their relatively high stability under alkaline conditions and promising activity for hydrogen evolution.^13–15,18,19^ Nevertheless, their hydrogen production activity is still insufficient, and further improvement in performance remains necessary.

In this context, improving the crystallinity and controlling the crystal structure are considered key strategies for enhancing the photocatalytic performance. In photocatalyst synthesis, metal–organic frameworks (MOFs),^20,21^ which are composed of metal cations and organic linkers, have attracted considerable attention as precursor materials. Their highly designable structures allow precise control over the composition, pore structure, and metal arrangement, making them advantageous platforms for the synthesis of functional materials. Moreover, this structural tunability can be extended to frameworks containing more than one type of metal cation. Owing to their well-defined compositions and ordered structures, MOFs have been widely utilized as precursors for the preparation of photocatalysts with a controlled morphology, composition, and crystal phase.^15,18,22,23^

In our previous studies, we employed MOFs as precursors for the synthesis of sulfide photocatalysts that were relatively stable under alkaline photoreforming conditions, and demonstrated that this approach improved the hydrogen evolution rate through enhanced crystallinity and crystal-structure control.^15,18^ Nevertheless, the overall hydrogen production activity remained insufficient. In the present study, to further improve hydrogen production, we focused on using a mixed-cation MOF as a precursor for the synthesis of solid-solution photocatalysts, based on the expectation that solid-solution formation would suppress charge recombination, facilitate charge separation, and thereby enhance the photocatalytic hydrogen evolution activity. Specifically, a Cd–Zn mixed MOF was synthesized as a precursor to prepare Cd_1–*x*_Zn_*x*_S solid-solution photocatalysts, with the aim of overcoming the intrinsic limitations of CdS photocatalysts. In addition, the photocatalytic performance of the MOF-derived solid-solution photocatalyst was compared with that of a Cd_1–*x*_Zn_*x*_S solid-solution photocatalyst without an MOF precursor. The hydrogen production performances of the prepared photocatalysts were evaluated through the photoreforming in the presence of cellulose, one of the major components of lignocellulosic biomass. Thus, we examined the effectiveness of mixed-cation MOF-derived synthesis to develop efficient photocatalysts for alkaline biomass photoreforming.

## Results and Discussion

A series of Cd–Zn mixed MOFs with different Cd/Zn ratios were synthesized using terephthalic acid as the organic linker, followed by sulfidation to obtain the corresponding sulfide photocatalysts. The actual metal composition of the resulting sulfides was determined by inductively coupled plasma (ICP) analysis. The measured Cd/Zn ratio of CdZnS-73 was 0.57:0.43, which deviated from the nominal feed ratio. To further investigate this discrepancy, ICP analysis was performed on the precursor MOF, CdZn-73-MOF. The Cd/Zn molar ratio was likewise determined to be 0.57:0.43, indicating that the Cd/Zn ratio of the precursor was preserved during sulfidation. The difference between the feed ratio and Cd/Zn ratio in the MOF is likely attributable to the differences in the ionic radius and hardness between Cd^2+^ and Zn^2+^. Because terephthalate is an O-donor ligand, Zn^2+^ is expected to form a more stable coordination environment, and hence, is considered to be incorporated into the crystal to a greater extent than that suggested by the initial feed ratio.

Figure 1(a) shows the X-ray diffraction (XRD) patterns of Cd-MOF, Zn-MOF, and CdZn-73-MOF, prepared with terephthalic acid as the organic linker and used as precursors for photocatalyst synthesis. Both Cd-MOF and CdZn-73-MOF exhibit characteristic peaks at approximately 9 and 18°, assigned to the (200) and (201) planes, respectively. The diffraction pattern of Cd-MOF is similar to a previously reported MOF pattern, suggesting the formation of a two-dimensional MOF-2 structure.^18,24^ CdZn-73-MOF shows the same characteristic peaks, with a slight shift toward higher diffraction angles. This shift suggests the incorporation of Zn^2+^, which has a smaller ionic radius than Cd^2+^, into the framework.^25^ The XRD pattern of Zn-MOF exhibits diffraction peaks assignable to the (200) and (220), suggesting that it adopts an MOF-5 structure.^26^

**Figure 1:**
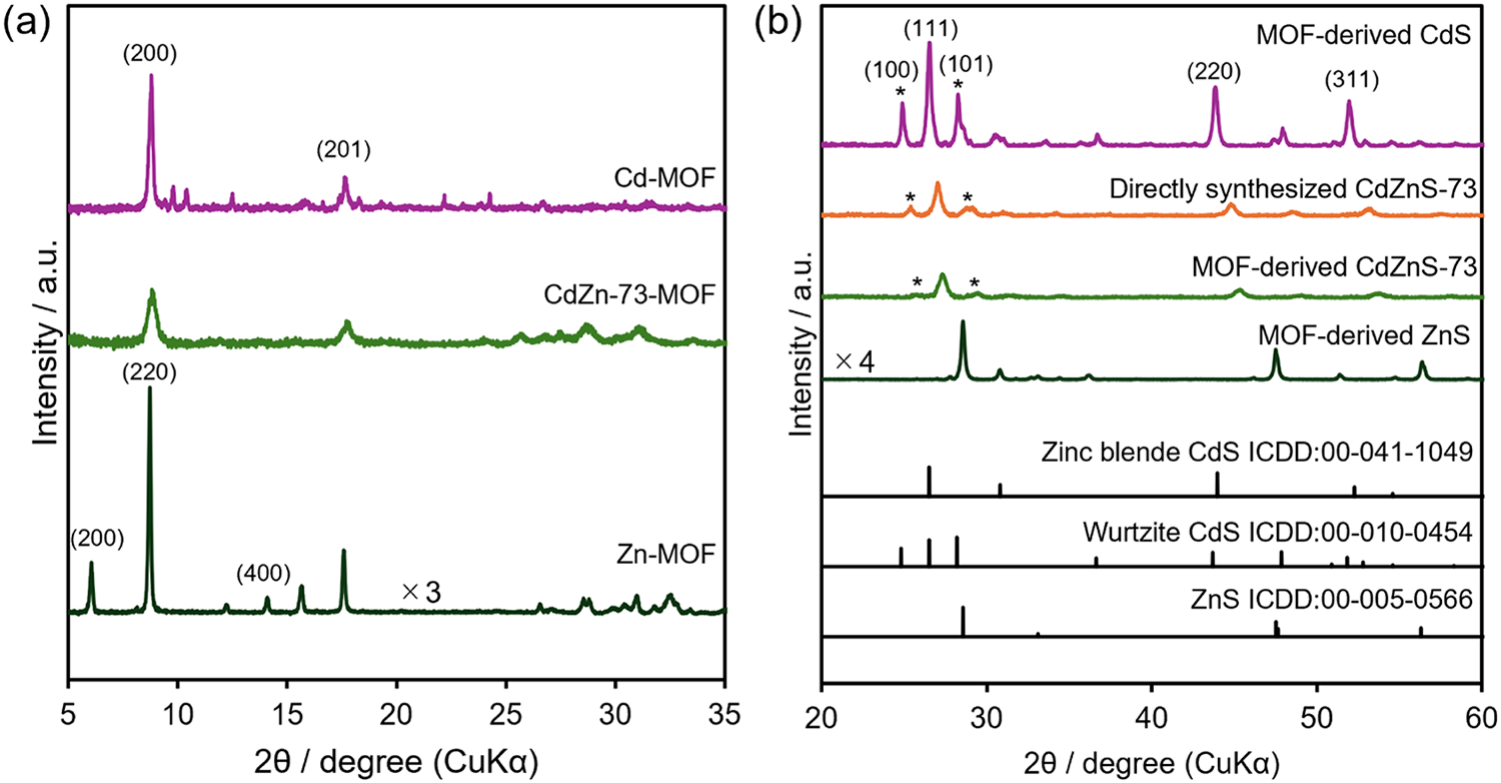
XRD patterns of (a) the terephthalic acid-based MOFs and (b) CdS, ZnS, and CdZnS-73 obtained by the sulfidation of the MOFs and the directly synthesized CdZnS-73 (peaks marked with * are from the wurtzite phase).

Figure 1(b) shows the XRD patterns of CdS, ZnS, and CdZnS-73 obtained by the sulfidation of the corresponding MOFs. These samples exhibit diffraction peaks assignable to the cubic phase, such as the (111), (220), and (311) reflections. For CdS, additional diffraction peaks associated with the (100) and (101) reflections, attributable to the wurtzite phase, are present. This indicates that the sample consists of mixed zinc blende and wurtzite phases, consistent with prior reports for sulfides synthesized from MOF precursors containing terephthalic acid as the organic linker.^18^ Meanwhile, CdZnS-73 obtained from the mixed-cation MOF precursor exhibits diffraction peaks that have shifted toward higher angles relative to those of CdS; for example, the peak at 27.3°, corresponding to the zinc blende (111) reflection, shifts from 26.5° in CdS. In the MOF-derived photocatalysts prepared with different Cd/Zn ratios, systematic shifts are observed with changes in the Cd/Zn ratio (Figure S1). This displacement suggests the formation of a homogeneous solid solution rather than a heterogeneous ZnS and CdS mixture.^27,28^ A weak diffraction peak attributable to the wurtzite phase is detected at approximately 26°, suggesting that the CdZnS-73 sample contains a minor wurtzite component. Rietveld refinement suggests a zinc blende-to-wurtzite ratio of 0.81:0.19 for the sample (Figure S2). Given the considerable peak broadening in the XRD patterns due to the nanosized particles and possible lattice strain, the phase compositions discussed above should be interpreted as approximate values derived from formal fitting.

For comparison, the XRD pattern of CdZnS-73 synthesized directly from Cd^2+^ and Zn^2+^ salts without using an MOF precursor was measured (Figure 1(b)). Focusing on the peak at 27.0° assigned to the (111) plane, a shift toward higher diffraction angles relative to CdS is observed, suggesting that both Cd^2+^ and Zn^2+^ are incorporated into the crystal lattice. The sample synthesized via the non-MOF route exhibits stronger wurtzite peaks relative to the zinc blende peaks than the sample prepared using the MOF precursor. Through Rietveld refinement, the zinc blende-to-wurtzite phase ratio was estimated to be 0.59:0.41 (Figure S2). In general, the wurtzite phase is regarded as a thermodynamically stable crystal structure at high temperatures for both CdS and ZnS, while wurtzite-type ZnS has also been reported to form under hydrothermal conditions below 200 °C, possibly because the solvent contributes to the stabilization of this metastable phase.^29^ A similar effect may have contributed to the formation of the wurtzite phase in the present study. By contrast, in the MOF-mediated synthesis process, hydrothermal treatment is accompanied by the decomposition of the MOF structure, which likely reduces the influence of the solvent and consequently favors the growth of zinc blende as the major phase.

Scanning electron microscopy (SEM) observations were performed to characterize the morphology of the synthesized MOF and the resulting solid solutions (Figure 2). Both CdZn-73-MOF and Cd-MOF exhibit sheet-like morphologies (Figure 2a,c), suggesting the possible formation of a two-dimensional MOF-2 structure, consistent with the XRD results. By comparison, Zn-MOF does not show a sheet-like morphology (Figure 2e). By contrast, the sulfidation product of the mixed-cation MOF CdZnS-73 (Figure 2b) exhibits particulate morphologies with sizes of approximately <100 nm, similar to those observed for MOF-derived CdS and ZnS (Figure 2d, f). These observations suggest that, while the morphology of the MOF precursor depends on the central metal species, the sulfide products synthesized via the solvothermal method lead to the formation of sulfide products with similar particulate morphologies. This can be attributed to Ostwald ripening during the transformation process. In addition, elemental mapping by EDS revealed that Cd, Zn, and S were distributed within the same particles (Figure S3, S4), suggesting that the product was present as Cd_1–*x*_Zn_*x*_S rather than as individual CdS and ZnS phases.

**Figure 2:**
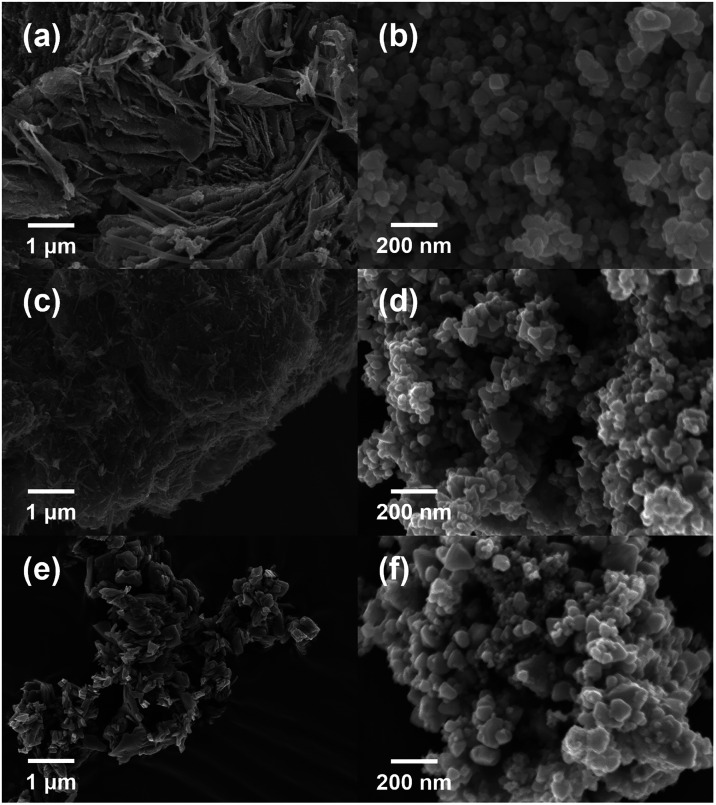
SEM images of (a) CdZn-73-MOF, (b) MOF-derived CdZnS-73, (c) Cd-MOF, (d) MOF-derived CdS, (e) Zn-MOF, and (f) MOF-derived ZnS.

Figure 3 shows the diffuse reflectance spectroscopy (DRS) spectra of the MOF-derived photocatalysts and the CdZnS**-**73 samples synthesized using various preparation methods. The absorption edges for the MOF-derived photocatalysts appeared sequentially for CdS, CdZnS**-**73, and ZnS moving from the longer-wavelength region. The optical band gaps were calculated from the Tauc plots in the same manner as in the study by Katayama et al.,^30^ where they were 2.30, 2.49, and 3.62 eV, respectively. These values align closely with previously reported band gap data for CdS and ZnS.^18,31,32^

**Figure 3:**
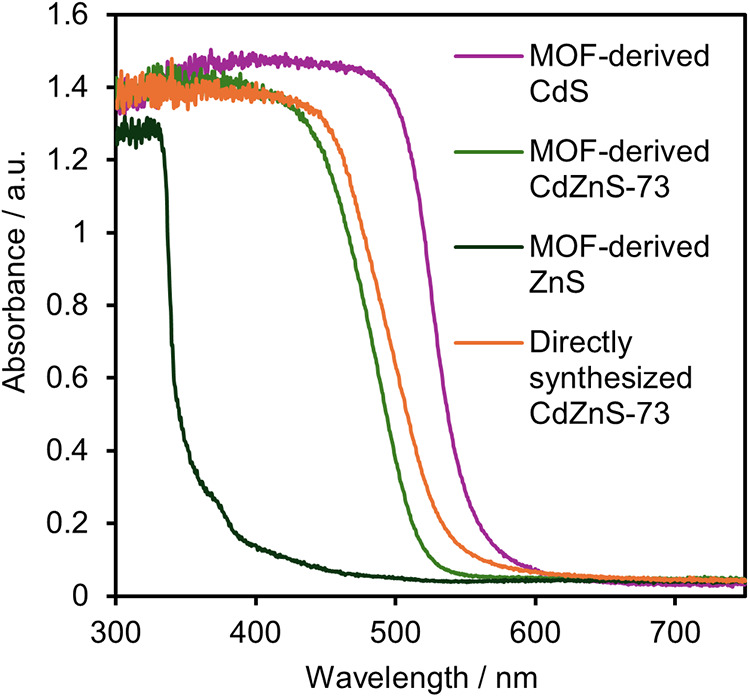
DRS spectra of the MOF-derived photocatalysts and the directly synthesized CdZnS-73.

In contrast, CdZnS**-**73 exhibited visible-light absorption characteristics. This behavior remains consistent with previously reported results, as the absorption spectrum is positioned between those of ZnS and CdS.^24^

The absorption edge for the CdZnS**-**73 sample synthesized without an MOF precursor shifted slightly toward the longer-wavelength region, as compared to the MOF-derived counterpart. The calculated band gap for this non-MOF-derived sample was 2.42 eV, representing a 0.07 eV reduction relative to that of the MOF-derived CdZnS**-**73.

To evaluate the potential practical applicability of the photocatalysts for photoreforming using cellulosic biomass, their hydrogen production activity was examined in an alkaline aqueous solution containing α-cellulose under simulated sunlight irradiation. Figure 4 shows the time course of hydrogen evolution and the corresponding hydrogen production rates. Compared with the MOF-derived CdS and ZnS samples, the Cd_1–*x*_Zn_*x*_S solid-solution photocatalysts exhibit a markedly enhanced hydrogen production activity (Figure S5). Comparison among the solid-solution photocatalysts prepared by the sulfidation of the MOF precursors synthesized with different Cd/Zn feed ratios revealed that CdZnS-73 exhibited the highest hydrogen production performance (Figure S5). It achieved a hydrogen production rate 7.2 times higher than that of CdS. For Cd_1–*x*_Zn_*x*_S solid solutions, it has been experimentally and theoretically reported that, as the Cd/Zn ratio decreases, the conduction-band edge shifts toward more negative potentials, while the valence-band edge shifts toward more positive potentials.^33,34^ In the present study as well, DRS measurements confirmed band gap narrowing in the solid-solution samples. These results suggest that the enhanced hydrogen production activity of CdZnS-73 can be attributed to an optimized narrowed band gap, a valence-band position that retains sufficient oxidation power for cellulose oxidation, and a conduction-band position that provides adequate reduction power for hydrogen evolution. In addition, CdZnS solid solutions have been reported to exhibit both direct and indirect transition characteristics,^35^ which may help suppress the recombination of photoexcited electrons and holes. This effect is also likely to contribute to the higher hydrogen production activity of CdZnS-73 than that of the MOF-derived CdS and ZnS samples. In addition, comparison of the XRD patterns before and after the photoreforming reaction revealed no changes, such as peak broadening or the appearance of new peaks, that have been reported to be associated with photocorrosion-induced etching or amorphization in alkaline solution,^36^ suggesting that CdZnS-73 was stable under the present photoreforming conditions (Figure S6).

**Figure 4:**
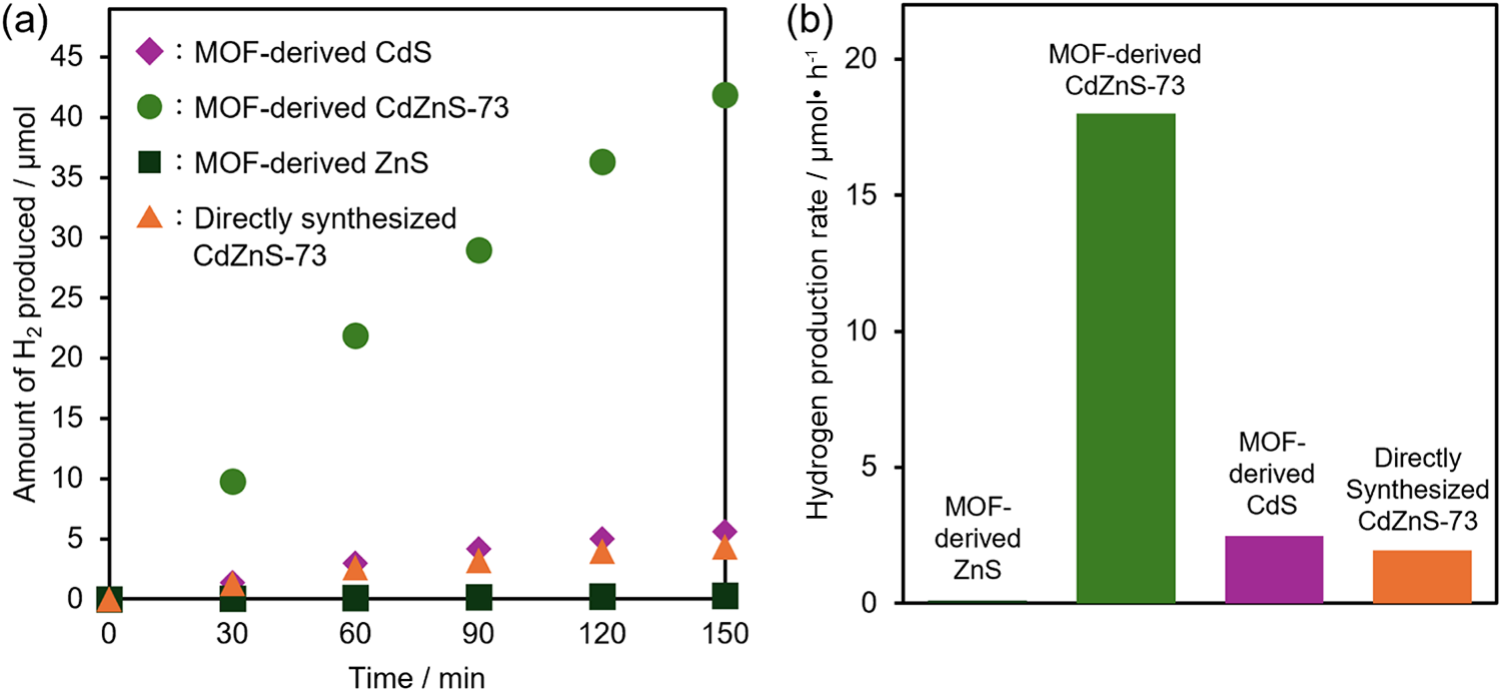
(a) Time course of the amount of evolved hydrogen over the MOF-derived photocatalysts and the directly synthesized CdZnS-73. (b) Hydrogen production rates calculated from (a).

Furthermore, the aforementioned MOF-derived CdZnS-73 exhibited a hydrogen evolution rate 9.2 times higher than that of the CdZnS sample synthesized without an MOF precursor (Figure 4). ICP analysis revealed that the directly synthesized CdZnS-73 sample had a Cd/Zn ratio of 0.73:0.27. Because the solid-solution compositions of the two samples were not identical and their band gaps estimated from DRS were also different, a strictly direct comparison is difficult. Nevertheless, the hydrogen production activity of the directly synthesized sample was lower even than that expected for an MOF-derived Cd_1–*x*_Zn_*x*_S photocatalyst with a comparable composition, suggesting a possible advantage of the MOF-mediated route. SEM observations showed that both samples consisted of similar nanoparticulate morphologies (Figures S3 and S4), implying that the differences in the particle shape or surface area were unlikely to be the primary origin of the observed difference in the activity. XRD analysis suggested that zinc blende was the dominant phase in MOF-derived CdZnS-73, while directly synthesized CdZnS-73 contained comparable amounts of the wurtzite and zinc blende phases. In general, the coexistence of multiple phases is often considered beneficial for the photocatalytic performance because type-II heterojunction formation can promote charge separation and suppress photoexcited electron and hole recombination.^37–39^ In the present study, however, the sample with the larger fraction of the wurtzite-phase content exhibited a lower hydrogen production activity, despite the generally accepted view that the wurtzite phase is more active in CdS photocatalysts. These observations imply that the phase composition alone is insufficient to account for the difference in the photocatalytic performance between the two samples. This prompted us to examine the solid-solution state in more detail, particularly by evaluating the composition dependence of the lattice constant in comparison with Vegard’s law. When the cubic-phase lattice constant *a* of the MOF-derived CdZnS-73 photocatalyst was compared with the value expected from its Cd/Zn ratio based on Vegard’s law (5.64 Å), the observed value (5.67 Å) showed a relatively large deviation. By contrast, the directly synthesized sample showed an *a* value of 5.71 Å, which was in agreement with the value expected from its Cd/Zn ratio based on Vegard’s law (5.71 Å). Such a deviation for MOF-derived CdZnS-73 suggests a departure from an ideal homogeneous solid solution and may reflect local compositional inhomogeneity, lattice strain, defects, or slight phase segregation. In other words, MOF-derived CdZnS-73 may possess a more structurally distorted and heterogeneous crystal lattice than CdZnS-73 synthesized without an MOF precursor. Previous studies have reported that structural disorder and defects can give rise to internal electric fields or dipole moments, thereby facilitating charge separation.^40,41^ Therefore, the difference in the photocatalytic performance observed in the present study may be associated not only with the average solid-solution composition but also with local structural distortion and heterogeneity introduced by the MOF-derived synthesis route.

## Conclusions

Cd–Zn sulfide solid-solution photocatalysts were synthesized in this study using MOF precursors to achieve stable and efficient hydrogen production via photoreforming. A series of solid-solution photocatalysts with different Cd/Zn ratios were successfully synthesized from Cd- and Zn-containing MOFs, among which CdZnS-73 exhibited a photocatalytic activity 7.2 times higher than that of pure CdS. Furthermore, compared with the sample synthesized without an MOF precursor, the MOF-derived sample showed 9.2 times higher photocatalytic activity despite possessing a similar solid-solution degree in terms of the Cd/Zn ratio. These results demonstrate that the use of MOFs as precursors is an effective synthetic strategy to prepare photocatalysts for the photoreforming of lignocellulosic biomass. Thus, the present MOF-derived solid-solution photocatalyst provides a promising approach for achieving efficient hydrogen production and advancing photocatalytic biomass conversion toward the resolution of global energy challenges.

## Methods

### Materials

Cd­(NO_4_)_3_·4H_2_O, Zn­(NO_4_)_3_·6H_2_O, triethylamine, N,N-dimethylformamide, and Na_2_S·9H_2_O were sourced from Kanto Chemical Co., Ltd. Terephthalic acid was purchased from Tokyo Chemical Industry Co., Ltd.

### Synthesis of the CdZnS-73 Solid-Solution Photocatalyst Using a MOF Precursor with Terephthalic Acid as the Organic Linker

The MOF-derived photocatalysts were synthesized using a methodology previously reported by Kamata et al.^13^ Specifically, 0.8 g of terephthalic acid and a mixture of 0.01 mol Cd­(NO_4_)_3_·4H_2_O (2.16 g, 0.007 mol) and Zn­(NO_4_)_3_·6H_2_O (0.89 g, 0.003 mol) were dissolved in 100 mL of N,N-dimethylformamide. Subsequently, 5.25 mL of triethylamine was added to the solution, which was stirred at room temperature for 2 h. The resulting white precipitate, designated as CdZn-73-MOF, was collected and washed with N,N-dimethylformamide. The Cd:Zn ratio was maintained according to the initial precursor loading. To obtain the final CdZnS-73 solid-solution photocatalyst, the resulting CdZn-73-MOF was dispersed in N,N-dimethylformamide and combined with 2.4 g of sodium sulfide dissolved in a minimal volume of pure water. The mixture was transferred to a Teflon-lined stainless-steel autoclave and heated at 120 °C for 24 h. Following natural cooling to room temperature, the resulting powder was washed with N,N-dimethylformamide, pure water, and methanol and subsequently dried at 70 °C for 24 h. Additional Cd_1–*x*_Zn_*x*_S samples with different compositions (0 ≤ *x* ≤ 1) were prepared by varying the Cd/Zn ratio under identical reaction conditions.

### Synthesis of the CdZnS-73 Solid-Solution Photocatalyst without a MOF Precursor

0.01 mol of Cd­(NO_4_)_3_·4H_2_O (2.16 g, 0.007 mol) and Zn­(NO_4_)_3_·6H_2_O (0.89 g, 0.003 mol) were dissolved in N,N-dimethylformamide. Following the addition of 2.4 g (0.01 mol) of sodium sulfide dissolved in a minimal volume of pure water, the mixture was transferred to a Teflon-lined stainless-steel autoclave and heated at 120 °C for 24 h. The cooling, washing, and drying protocols remained identical to those utilized for the MOF-derived samples.

### Characterization

The prepared Cd_1–x_Zn_*x*_-MOF and Cd_1–*x*_Zn_*x*_S solid-solution photocatalyst samples were characterized using XRD (SmartLab, Rigaku, Tokyo, Japan), DRS (U-3900/3900H spectrophotometer, Hitachi High-Tech Science, Tokyo, Japan), SEM-EDS (GeminiSEM 360, Carl Zeiss GmbH, Oberkochen, Germany), and ICP–OES (SPECTRO ARCOS MV130, SPECTRO Analytical Instruments GmbH, Kleve, Germany).

### Photocatalytic Activity Evaluation

Hydrogen production experiments were conducted in 32 mL test tubes sealed with rubber stoppers under an argon atmosphere. Each test tube contained 50 mg of the prepared photocatalyst, 5 mL of a 10 M NaOH reaction solution, and 100 mg of α-cellulose (38 μm mesh-through powder, Wako Pure Chemical Industries, Ltd.) as the substrate. The suspension was stirred at a constant temperature of 70 °C in a thermostatic bath under side-irradiation from an AM1.5 solar simulator. The evolved gases were sampled using a gastight syringe and analyzed via gas chromatography (GC-8 A, Shimadzu Corporation) to quantify the hydrogen production. The hydrogen production rate was determined based on the total yield obtained within a 150 min reaction interval.

## Supplementary Material



## Notes

XRD patterns (Figures S1, S2, and S3); SEM images and EDS mapping (Figures S3 and S4); and time course of the amount of evolved hydrogen and hydrogen production (Figure S5) (PDF)
