# Supplementary material for: Design of MOF-Derived Cd1–x Zn x S Solid-Solution Photocatalysts and Evaluation of Hydrogen Production by Photoreforming
Source: ACS Omega. 2026 May 18;11(21):31477–83. doi: 10.1021/acsomega.6c01944 (PMC13234805; doi:10.1021/acsomega.6c01944)
Supplement: Supplementary file 1 [file ao6c01944_si_001.pdf]

Supporting Information

Design of MOF-derived $Cd_{1-x}Zn_xS$ solid solution photocatalysts and evaluation of hydrogen production by photoreforming

Yusei Kumai[†], Yuya Takekuma[‡], and Morio Nagata*[†,‡]

†Department of Industrial Chemistry, Graduate School of Engineering, Tokyo University of Science, 6-3-1 Niijuku, Katsushika-ku, Tokyo 125-8585, Japan

‡Department of Industrial Chemistry, Faculty of Engineering, Tokyo University of Science, 6-3-1 Niijuku, Katsushika-ku, Tokyo 125-8585, Japan

Hydrogen Production, Photoreforming, Photocatalyst, Solid Solution, Metal-Organic framework

E-mail: nagata@ci.tus.ac.jp

Supplementary Figures

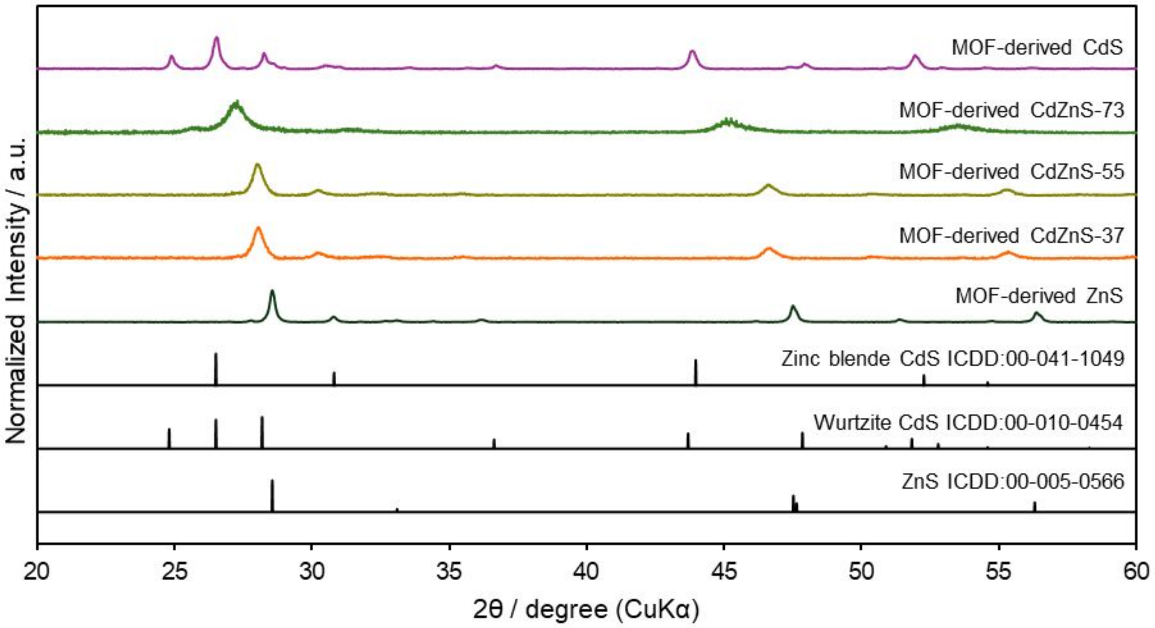

Figure S1. XRD patterns of CdS, ZnS, and various CdZnS obtained by the sulfidation of the corresponding MOFs.

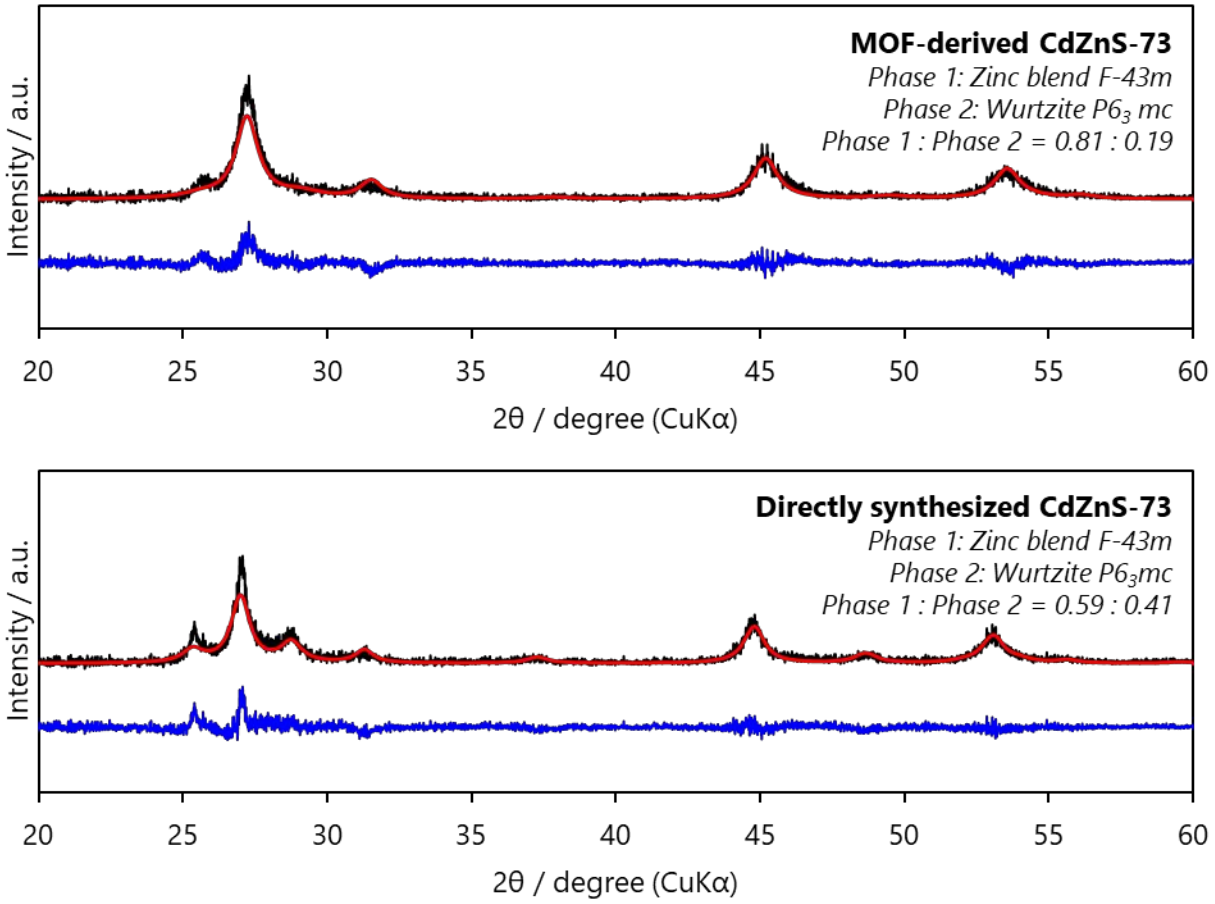

Figure S2. XRD patterns of CdZnS-73 derived MOF and synthesized directly in which observed (black), calculated (red), and differences (blue).

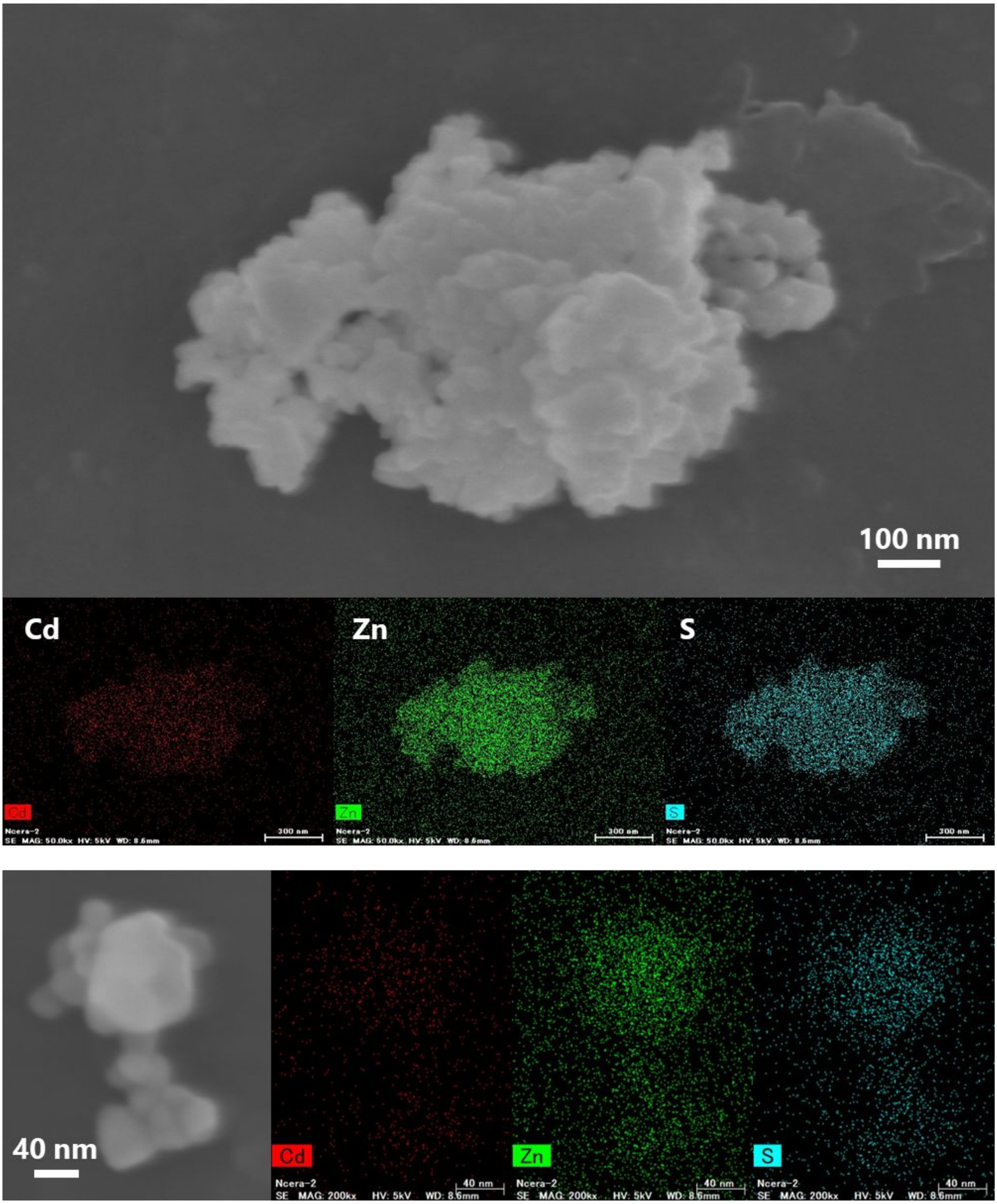

Figure S3. SEM images and EDS mapping of the MOF-derived CdZnS-73.

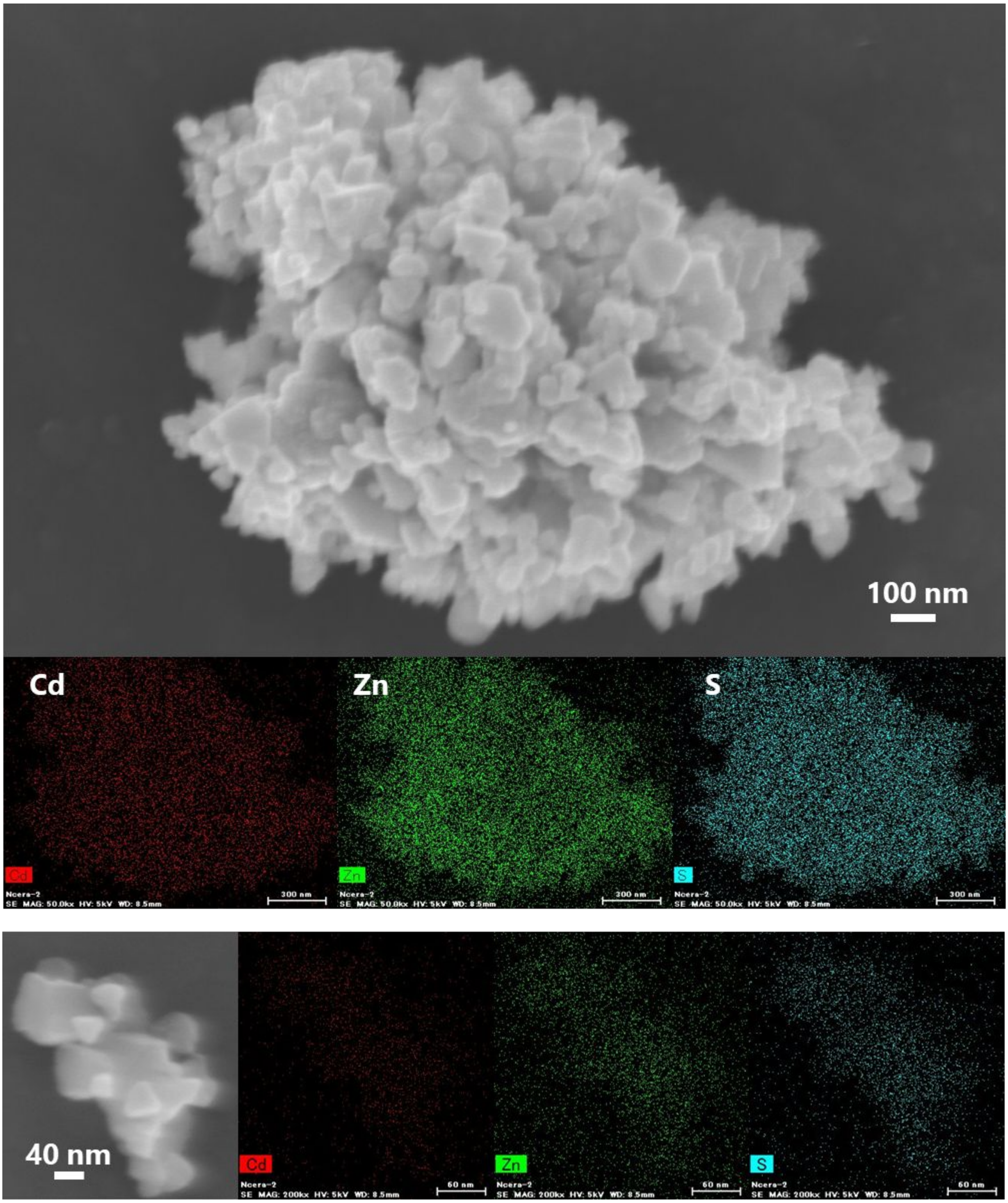

Figure S4. SEM images and EDS mapping of the directly synthesized CdZnS-73.

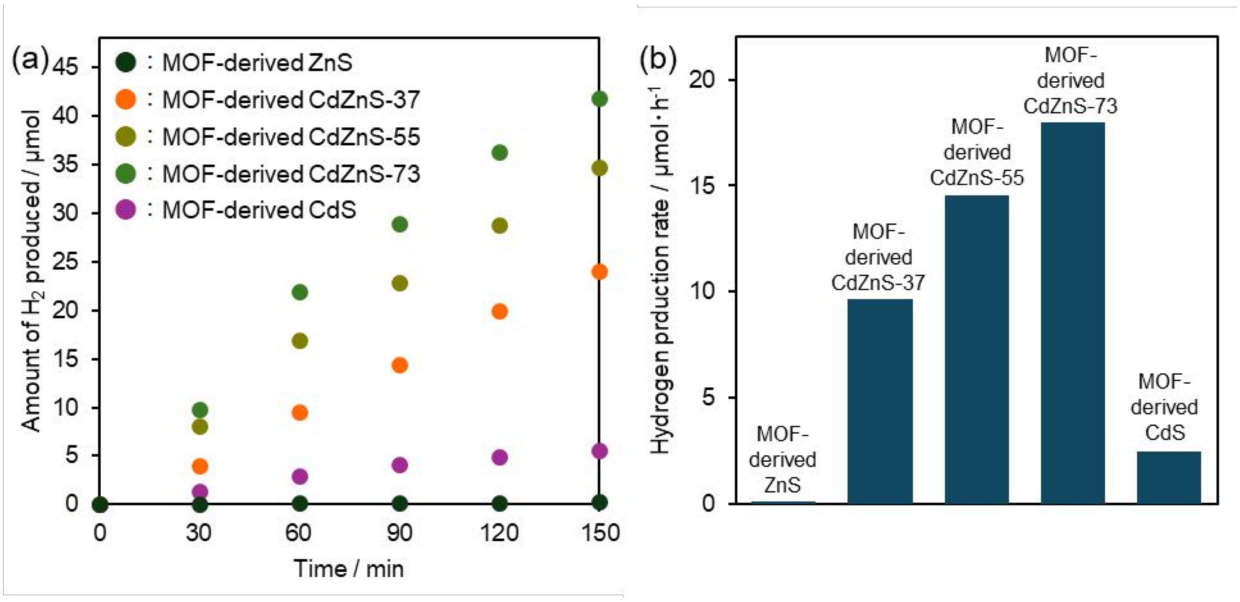

Figure S5. (a) Time course of the amount of evolved hydrogen over the various MOF-derived photocatalysts. (b) hydrogen production rates calculated (a).

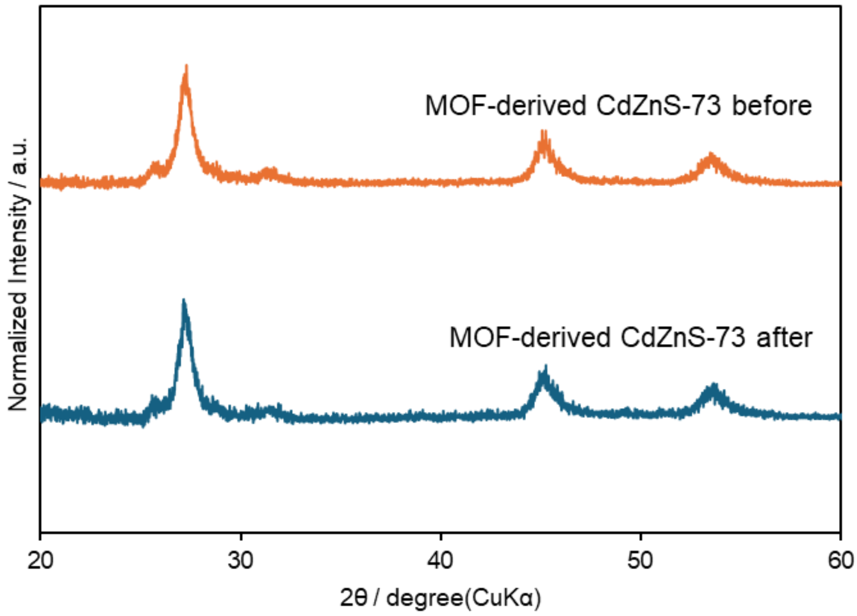

Figure S6. XRD patterns of the MOF-derived CdZnS-73 before and after the photoreforming reaction.
